# *In Vivo* Time-Course Imaging of Tumor Angiogenesis in Colorectal Liver Metastases in the Same Living Mice Using Two-Photon Laser Scanning Microscopy

**DOI:** 10.1155/2012/265487

**Published:** 2011-11-03

**Authors:** Koji Tanaka, Yuhki Morimoto, Yuji Toiyama, Kohei Matsushita, Mikio Kawamura, Yuhki Koike, Yoshinaga Okugawa, Yasuhiro Inoue, Keiichi Uchida, Toshimitsu Araki, Akira Mizoguchi, Masato Kusunoki

**Affiliations:** ^1^Department of Gastrointestinal and Pediatric Surgery, Mie University Graduate School of Medicine, 2-174 Edobashi, Tsu, Mie 514-8507, Japan; ^2^Department of Neural Regeneration and Cell Communication, Mie University Graduate School of Medicine, 2-174 Edobashi, Tsu, Mie 514-8507, Japan

## Abstract

*In vivo* real-time visualization of the process of angiogenesis in secondary tumors in the same living animals presents a major challenge in metastasis research. We developed a technique for intravital imaging of colorectal liver metastasis development in live mice using two-photon laser scanning microscopy (TPLSM). We also developed time-series TPLSM in which intravital TPLSM procedures were performed several times over periods of days to months. Red fluorescent protein-expressing colorectal cancer cells were inoculated into the spleens of green fluorescent protein-expressing mice. First- and second-round intravital TPLSM allowed visualization of viable cancer cells (red) in hepatic sinusoids or the space of Disse. Third-round intravital TPLSM demonstrated liver metastatic colonies consisting of viable cancer cells and surrounding stroma with tumor vessels (green). *In vivo* time-course imaging of tumor angiogenesis in the same living mice using time-series TPLSM could be an ideal tool for antiangiogenic drug evaluation, reducing the effects of interindividual variation.

## 1. Introduction

Angiogenesis is a fundamental process for the production of new blood vessels during reproduction, embryonic development, and wound healing [1]. It is also a hallmark of tumor growth and metastasis, and high levels of tumor angiogenesis are associated with advanced tumor growth, distant metastases, and an adverse prognosis in human cancers, including colorectal cancer (CRC) [2]. 

CRC is the second most frequent cause of cancer-related deaths worldwide. Although significant progress in the treatment of metastatic CRC has increased the median overall survival (OS) to around 24 months [3, 4], the 5-year OS in patients with stage IV CRC with liver metastasis remains below 10%, despite intensive multidisciplinary therapies [5]. There is therefore an urgent need to understand the mechanisms of colorectal liver metastasis and tumor angiogenesis. 

Multiphoton microscopy, including two-photon laser scanning microscopy (TPLSM), has been introduced to tumor biology during the last decade and has become a common instrument in the biological laboratory [6–8]. We have established a new method for *in vivo* real-time TPLSM imaging of intra-abdominal gastrointestinal disease using green-fluorescent-protein- (GFP-) expressing mice [9].

*In vivo* real-time TPLSM imaging of colorectal liver metastasis formation is achieved by inoculating red-fluorescent-protein- (RFP-) expressing cell lines into the spleens of GFP mice. This involves fixation of the liver using an organ-stabilizing system to minimize microvibration of the observed area caused by heart beat and respiratory movements, thus allowing the liver to be visualized at higher magnifications in the living mice.

We also established a time-series TPLSM technique consisting of several intravital TPLSM observations at different time points over prolonged experimental periods to allow the dynamics of liver metastasis formation to be followed in the same living mice over periods of months. 

In this study, *in vivo* real-time dual-color imaging of colorectal liver metastasis formation with tumor angiogenesis was performed using intravital and time-series TPLSM.

## 2. Materials and Methods

### 2.1. Animals

GFP-expressing nude mice (C57BL/6-BALB/c-nu/nu-EGFP) were purchased from AntiCancer Japan (Osaka, Japan). GFP nude mice (20–22 g) were bred, housed in groups of six mice per cage, and fed with a pelleted basal diet (CE-7, CLEA Japan Inc., Tokyo, Japan). Mice had free access to drinking water. They were kept in the animal house facilities at Mie University School of Medicine under standard conditions of humidity (50 ± 10%), temperature (23 ± 2°C), and light (12/12 h light/dark cycle), according to the Institutional Animal Care Guidelines. The experimental protocols were reviewed and approved by the Animal Care and Use Committee at Mie University Graduate School of Medicine.

### 2.2. Human CRC Cell Line

The RFP-expressing human CRC cell line (RFP-HT29) was purchased from AntiCancer Japan. RFP-HT29 cells were grown in monolayer cultures in RPMI 1640 (Sigma-Aldrich, Inc., St. Louis, Mo, USA) supplemented with fetal bovine serum (10% (v/v), GIBCO BRL, Tokyo, Japan), glutamine (2 mM), penicillin (100,000 units/L), streptomycin (100 mg/L), and gentamycin (40 mg/L) at 37°C in a 5% CO_2_ environment. For routine passage, cultures were spilt 1 : 10 when they reached 90% confluence, generally every 3 days. Cells at the fifth to ninth passage were used for liver metastasis experiments.

### 2.3. Experimental Liver Metastasis Model

RFP-HT29 cells were inoculated into the spleens of GFP nude mice, as a xenogeneic tumor model. RFP-HT29 cells at the fifth to ninth passage were harvested with trypsin/EDTA and washed in serum-containing RPMI 1640 medium to inactivate any remaining trypsin. The cells were centrifuged and resuspended in phosphate-buffered saline (PBS). Finally, the cells were adjusted to 1 × 10^7^ cells/mL for single-cell suspensions. GFP nude mice were anesthetized by intraperitoneal injection of chloral hydrate (Sigma, St Louis, Mo, USA). Under direct vision, 1 × 10^6^ cells were injected into the spleen using a 30-gauge needle through a small incision in the left lateral abdomen of anesthetized GFP nude mice.

### 2.4. Surgical Procedures for Intravital TPLSM (Figure 1)

After inoculation, GFP nude mice were anesthetized by intraperitoneal injection of chloral hydrate. Body temperature was kept at 37°C throughout the experiments using a heating pad. The upper midline laparotomy was made as short as possible (<15 mm). The left lateral lobe of the liver was identified and exteriorized through the laparotomy. The liver lobe was then put onto an organ-stabilizing system (Japanese Patent Application number; P2007-129723) using a solder lug terminal with an instant adhesive agent (KO-10-p20, DAISO, Japan). The organ stabilizer minimized the microvibration of the observed area caused by heart beat and respiratory movements. Stabilization and fixation of the liver lobe represented a critical but technically difficult part of the intravital TPLSM procedure. After the application of PBS to the observed area, a thin cover glass was placed gently on the liver surface. After intravital TPLSM, the exteriorized liver lobe was gently removed from the organ-stabilizing system using a release agent (KO-10-p8, Daiso, Japan), to prevent liver injury. A sodium hyaluronate and carboxymethylcellulose membrane (Seprafilm Adhesion Barrier, Genzyme Corporation, Cambridge, Mass) was placed between the liver and the abdominal wall to prevent postoperative dense adhesion.

### 2.5. Time-Series TPLSM for Time-Course Imaging

The process of tumor angiogenesis during colorectal liver metastasis was observed in the livers of the same living mice repeating TPLSM at multiple time points using the above-mentioned surgical procedures until nondissecting adhesions formed between the liver and the abdominal wall. In preliminary experiments, the intravital TPLSM images at four or more time points were too unclear to observe colorectal liver metastases. If there was no dense fibrous adhesion between the liver and the abdominal wall, intravital TPLSM with clear image could be performed four or more times over intervals ranging from days to months. In reality, the time-series TPLSM consisted of three times intravital TPLSM procedures performed on the same mouse (see below; Section 2.8). The intervals of three time points (2 hour, 24 hour, and 8 week after inoculation) were also determined by preliminary experiments before this study. Precautions to prevent postoperative intraperitoneal infection were taken during the entire surgical procedure of time-series TPLSM.

### 2.6. TPLSM Setup

The procedures for TPLSM setup were performed as previously described [9]. Experiments were performed using an upright microscope (BX61WI; Olympus, Tokyo, Japan) and a FV1000-2P laser-scanning microscope system (Fluoview FV1000MPE, Olympus, Tokyo, Japan). The use of special stage risers enabled the unit to have an exceptionally wide working distance. This permitted the stereotactically immobilized, anesthetized mouse to be placed on the microscope stage. The microscope was fitted with several lenses with high numeric apertures to provide the long working distances required for *in vivo *work and with water-immersion optics. The excitation source in TPLSM mode was Mai Tai Ti: sapphire lasers (Spectra Physics, Mountain View, Calif), tuned, and mode-locked at 910 nm. The Mai Tai produces light pulses of about 100 fs width (repetition rate 80 MHz). Laser light reached the sample through the microscope objectives, connected to an upright microscope (BX61WI; Olympus, Tokyo, Japan). A mean laser power at the sample was between 10 and 40 mW, depending on the depth of imaging. Microscope objective lenses used in this study were 4x UPlanSApo (numerical aperture of 0.16), 10x UPlanSApo (numerical aperture of 0.4), and 60x LUMPlanFI/IR (water dipping, numerical aperture of 0.9, working distance 2 mm), respectively. Data were analyzed using a FV10-ASW (Olympus, Tokyo, Japan). TPLSM images were acquired with 512 × 512 pixels spatial resolution, from 210 *μ*m field of view dimension, using a pixel dwelling time 4 *μ*s. Two-photon fluorescence signals were collected by an internal detector (nondescanned detection method) at an excitation wavelength, to enable the simultaneous acquisition of EGFP signal and RFP (DsRed2) signal. In fact, an excitation wavelength of 1050 nm is optimum for DsRed2 as reported by Kawano et al. [10], although an excitation wavelength of 910 nm is optimum for EGFP. Therefore, it is difficult to excite DsRed2 at 910 nm amply. To overcome this difficulty, we have selected and used the tumor cells in which expression level of DsRed2 is so high that we can identify the DsRed2-labeled tumor cells clearly even with the 910 nm excitation. Color-coded green and red images were imaged at the same time and subsequently merged to produce single images.

### 2.7. Imaging of Colorectal Liver Metastasis Using Interval TPLSM

The surface of the liver lobe was initially screened at lower magnifications by setting out the *X/Y* plane and adjusting the *Z* axis manually to detect the optimal observation area containing RFP-expressing cancer cells (at least five areas). Each area of interest was subsequently scanned at a higher magnification (water-immersion objective 60x with or without 2x zoom) by manually setting the *X/Y* plane and adjusting the *Z* axis (either automatically or manually) to obtain high-resolution, clear TPLSM images. The scanning areas were 200 × 200 *μ*m (600x) or 100 × 100 *μ*m (600x with 2x zoom), respectively. The imaging depth or imaging stack was determined arbitrarily to allow real-time three-dimensional visualization of colorectal liver metastasis *in vivo*. The laser power was adjusted according to the imaging depth. When imaging at larger depths, we increase the laser-power level (up to 100%) manually using laser power level controller. To image the optimal simultaneous imaging of EGFP and RFP (DsRed2), detection sensitivity (brightness by HV) was adjusted manually for EGFP (450–500) or RFP (550–600), respectively.

### 2.8. Experimental Schedule for Time-Series TPLSM

RFP-HT29 cells were inoculated into the spleens of GFP nude mice. Colorectal liver metastasis formation was observed by 8 weeks in this xenogeneic tumor model (data not shown). As shown in Figure 2, first-round intravital TPLSM was performed to observe the early stages of colorectal liver metastasis (tumor cell arrest/adhesion and tumor-cell-induced platelet aggregation) at 2 hours after inoculation. Second-round intravital TPLSM was also performed to observe the early stages of colorectal liver metastasis (Phagocytosis by Kupffer cells and extravasation of cancer cells) at 24 hours after inoculation in the same mouse. Established liver metastases were observed during third-round intravital TPLSM, performed 8 weeks after the inoculation of RFP-HT29 cells into GFP nude mice. With regard to these time points (2 hour, 24 hour, and 8 week after inoculation), we have performed preliminary experiments to determine the optimal time points. The intravital TPLSM images at four or more time points were too unclear to observe colorectal liver metastases because of abdominal fibrous adhesion. Thus, all mice were imaged at three time points. At the end of the experiments (8 weeks after inoculation), the whole liver was harvested and subjected to histopathological analysis.

### 2.9. Immunohistochemical Analysis of Cytokeratin 20

The lack of immunological cross-reactivity with other cytokeratins means that CK 20 has become an important tool for delineating the origin of metastatic human adenocarcinomas arising from an unknown primary source. Mouse livers were removed and fixed in 4% formaldehyde in PBS (pH 7.4) for 24 hours, processed, and embedded in paraffin wax according to standard procedures. Formalin-fixed, paraffin-embedded tissue was sliced at a thickness of 3 *μ*m, and the sections were placed on silane-coated slides. After deparaffinization and dehydration, the sections were autoclaved for 10 minutes in 10 mM sodium citrate buffer for antigen retrieval. They were blocked and incubated with primary antibody overnight at 4°C. Primary monoclonal anti-human cytokeratin 20 antibody (Clone KBsB20.8; DakoCytomation, Denmark) was used at a dilution of 1 : 50 for implementation of the labeled streptavidin-biotin method (LASB2 kit/HRP, DakoCytomation). Cytokeratin 20 was detected using Envision reagents (Envision kit/HRP, DakoCytomation). The sections were counterstained using hematoxylin. Negative controls were run simultaneously with preimmune immunoglobulin.

## 3. Results

### 3.1. The Development of Intravital TPLSM for *In Vivo* Real-Time Imaging of Colorectal Liver Metastasis

**Figure 1:**
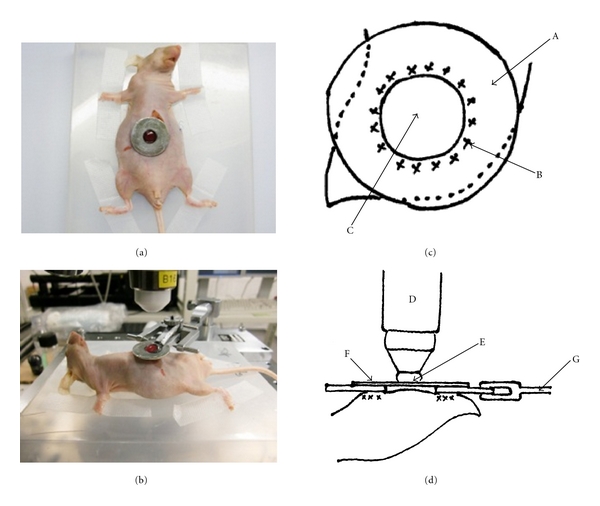
Overview and schematic drawing of liver-lobe fixation and intravital TPLSM setup. (a) Exteriorization of the left lateral lobe following upper midline laparotomy, followed by fixation of the left lateral lobe to a solder lug terminal using an instant adhesive agent. (b) Placement of an anesthetized mouse on the stage and setup of the organ-stabilizing system. (c) A solder lug terminal was adhered in a waterproof manner to the surface of the left lateral liver lobe using an instant adhesive agent. (d) PBS was applied to the observation area, and a watertight thin cover glass was placed over the area. The solder lug terminal was held on by the organ-stabilizing system. A: solder lug terminal, B: instant adhesive agent, C: observation area, D: objective, E: water immersion, F: cover glass, G: organ-stabilizing system.

**Figure 2:**
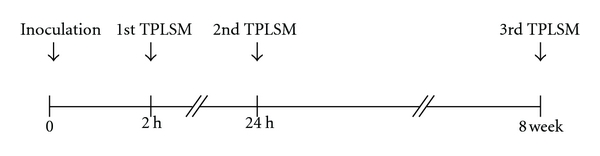
Experimental protocol and timing of intravital TPLSM. First-round intravital TPLSM at 2 hours after inoculation. Second-round intravital TPLSM at 24 hours after inoculation in the same mouse, for imaging the early events of colorectal liver metastasis. Third-round intravital TPLSM at 8 weeks after inoculation in the same mouse, for imaging-established liver metastases and tumor angiogenesis.

Figure 1 shows an overview and schematic drawing of liver-lobe fixation and intravital TPLSM setups. 

The key steps were (1) optimal longitudinal laparotomy; (2) choice of left lateral lobe of the liver for fixation using an organ-stabilizing system; (3) liver fixation using a solder lug terminal and an instant adhesive agent; (4) adjustment of detector gain for simultaneous dual-color imaging of RFP-expressing cells in the liver of GFP mice; (5) release of the liver lobe from the solder lug terminal using a release agent and placement of a Seprafilm Adhesion Barrier to the abdomen for interval TPLSM. 

A success rate approaching 100% for intravital and interval TPLSM can be achieved after a practice period.

### 3.2. Imaging of Early Colorectal Liver Metastasis by Intravital TPLSM

Intravital TPLSM imaging can be represented as a time-lapse two-dimensional movie and also as a *z*-stack three-dimensional movie from the liver surface to approximately 100–200 *μ*m depth. The imaging depth for three-dimensional visualization of the *in vivo* real-time metastatic events and tumor angiogenesis was determined arbitrarily and depended in part on the positioning of the liver lobe using the organ-stabilizing system or on the laser power. 

To observe the early events of colorectal liver metastasis at higher resolution and in dual color, at least five areas containing RFP-expressing cancer cells were initially identified at lower magnifications. Each area of interest was subsequently observed at higher magnification. The scanning areas were 200 × 200 *μ*m or 100 × 100 *μ*m, respectively. If necessary, additional five areas were observed by manually setting the *X/Y* plane and either automatically or manually adjusting the *Z* axis. 

**Figure 3:**
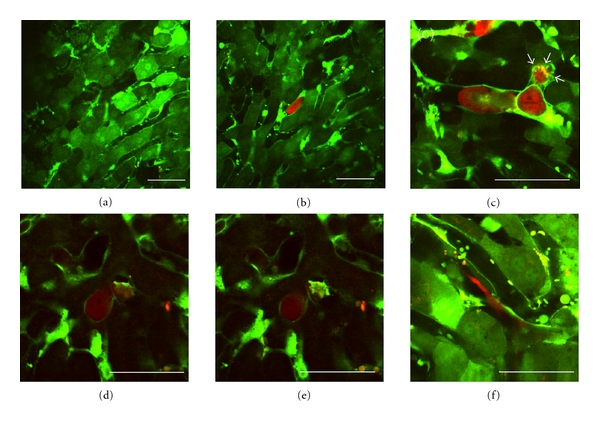
Imaging of early events of colorectal liver metastasis by intravital TPLSM. (a) Normal liver. (b) Arrest of a tumor cell in the hepatic sinusoid (2 hours after inoculation). (c) Tumor-cell-induced platelet aggregation (white arrows, 2 hours after inoculation). (d) Phagocytosis by a Kupffer cell (24 hours after inoculation). (e) Phagocytosis by a Kupffer cell at different depth. (f) Extravasation of a tumor cell (24 hours after inoculation) (bar, 50 *μ*m).

Table 1 shows the incidence of the early events of colorectal liver metastasis using this model. The phenomena of tumor cell arrest/adhesion and tumor-cell-induced platelet aggregation [11, 12] were frequently observed at 2 hours after inoculation (Figure 3(b), 3(c)). Phagocytosis by Kupffer cells and extravasation of cancer cells were observed within 24 hours after inoculation (Figure 3(d), 3(e), 3(f)) but were rare events.

### 3.3. Imaging of Liver Metastatic Colonization and Tumor Angiogenesis by Intravital TPLSM

One mouse died of unknown causes within 24 hours of inoculation. Second-round intravital TPLSM was performed successfully in the remaining 19 GFP nude mice (19/19; 100%). Two more mice had died of colorectal liver metastases by 8 weeks after inoculation, before the scheduled third-round intravital TPLSM. The remaining 17 GFP nude mice were successfully imaged by third-round intravital TPLSM (17/17; 100%). 

Among these 17 GFP nude mice, 12 (70%) had liver metastases with localized growth patterns at 8 weeks after inoculation. Only the left lateral lobe of the liver could be imaged using our experimental protocol, and four mice had liver metastases outside the observation area. As a result, only eight (67%) of the 12 mice were successfully imaged by third-round intravital TPLSM (Table 2). 

**Figure 4:**
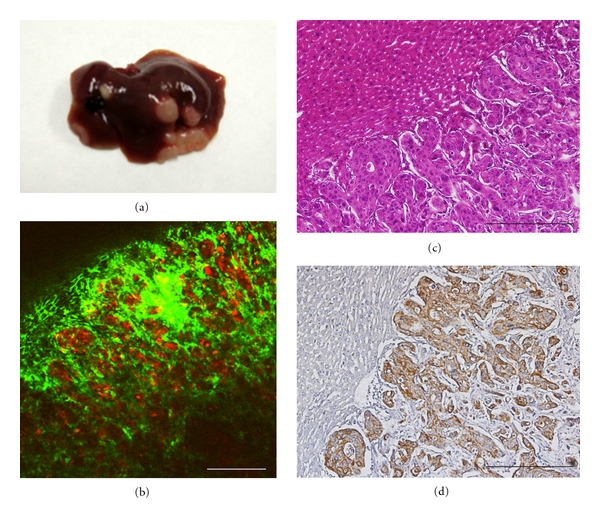
Liver metastatic colonization by RFP-expressing HT29 cells. (a) Macroscopic findings. (b) Intravital TPLSM image (bar, 300 *μ*m). (c) Microscopic hematoxylin-eosin staining (bar, 500 *μ*m). (d) Microscopic cytokeratin 20 immunostaining (bar, 500 *μ*m).

Several metastatic nodules had been formed by RFP-HT29 cells approximately 8 weeks later (Figure 4(a), 4(b), 4(c)). Anti-human cytokeratin 20 antibody was used for the conformation of RFP-HT29 cells in the xenogeneic liver metastasis model. RFP-HT29 cells in micrometastatic colonies (brown colored cells) were shown immunohistochemically (Figure 4(d)). 

**Figure 5:**
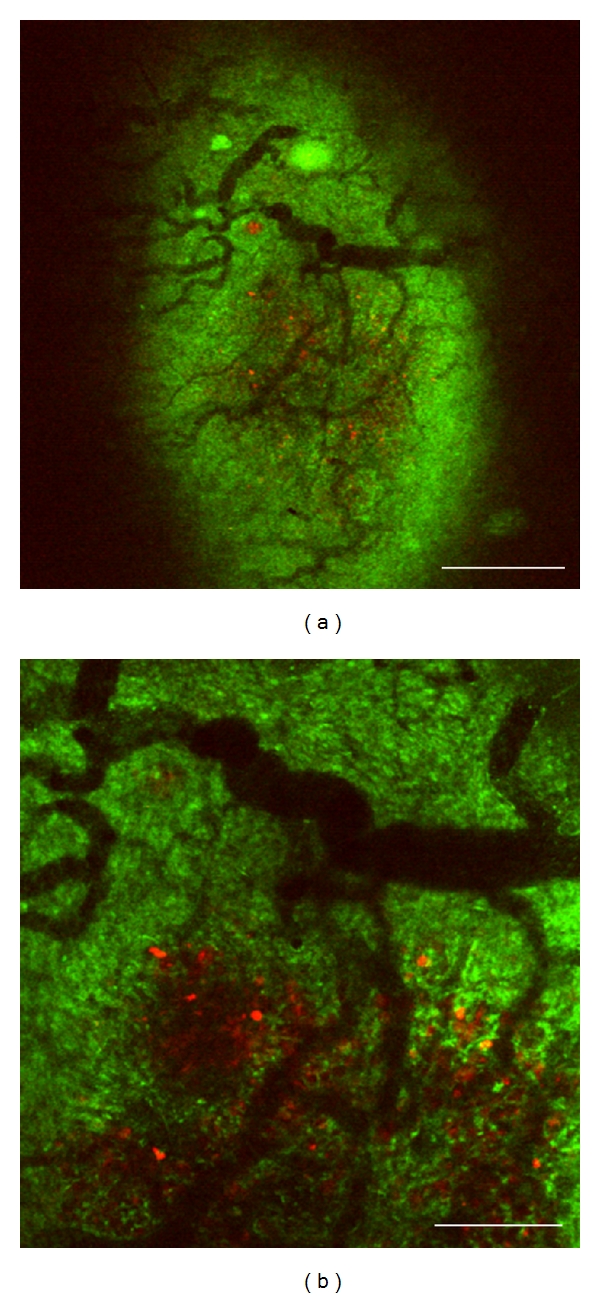
Imaging of liver metastasis and tumor angiogenesis by intravital TPLSM. (a) Liver metastatic nodule with dilated and tortuous tumor vessels (×40, bar, 750 *μ*m); (b) (×100, bar, 300 *μ*m).

**Figure 6:**
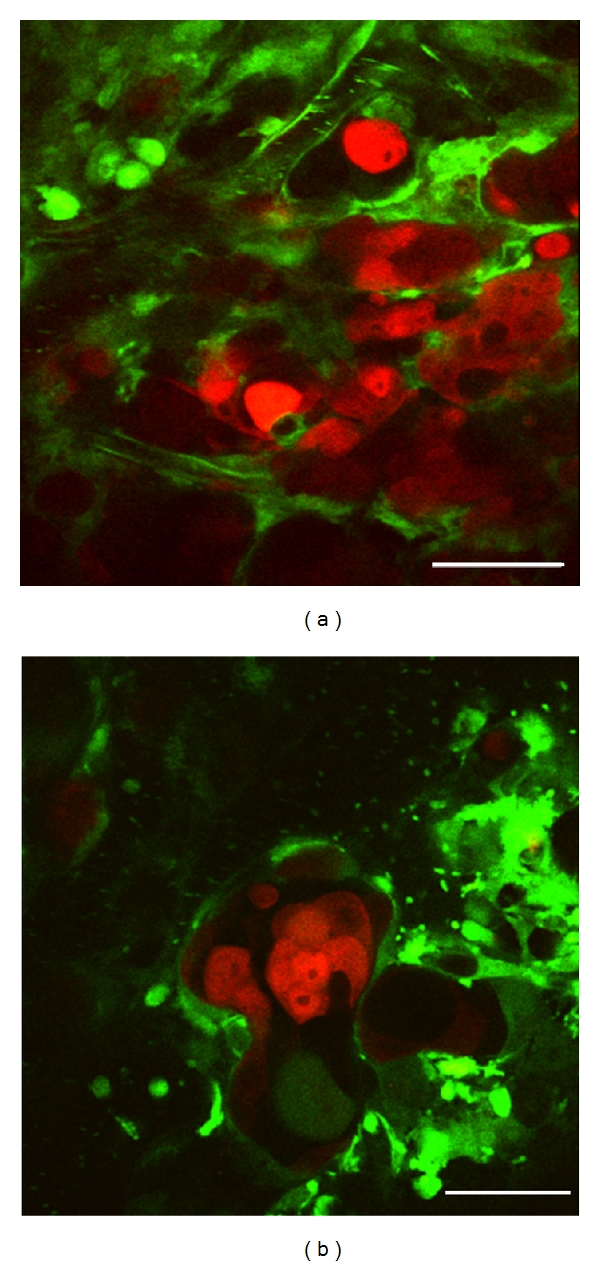
High-resolution optical imaging of liver metastatic nodule by intravital TPLSM. (a) Liver metastatic nodules were composed of tumor cell clusters and dilated/tortuous vessels (×600, bar, 50 *μ*m). (b) Dilated and tortuous tumor vessels were observed among the clusters of several tumor cells. A flow of aggregated platelets was frequently observed within the tumor vessels (×600, bar, 50 *μ*m).

Each nodule was composed of tumor cell clusters and dilated/tortuous tumor vessels (Figures 5, and 6). A flow of aggregated platelets was frequently observed within the tumor vessels, suggesting the existence of a hypercoagulable state in the tumor microenvironment (data not shown).

## 4. Discussion and Conclusion

We have established a new method for investigating tumor metastasis and angiogenesis using intravital TPLSM. Using our model, the real-time development of colorectal liver metastases can be imaged *in vivo* at high optical resolution and in dual color (red: cancer cells, green: host cells) using intravital TPLSM. It is also possible to perform time-course imaging of metastasis and angiogenesis in the livers of the same living mice using time-series TPLSM, which consists of three times intravital TPLSM procedures performed at different time points over periods of days to months. 

These techniques enabled us to observe metastatic nodules containing viable cancer cells (red) and tumor vessels in the surrounding stroma (green) in the same living mice at different time points. 

In this study, red-colored cancer cells were visualized in real-time in the green-colored liver structures of GFP mice *in vivo*. Tumor cell arrest/adhesion, tumor-cell-induced platelet aggregation, tumor cell extravasation, and phagocytosis by Kupffer cells could be observed using both time-lapse and *z*-stack imaging techniques (data not shown). Liver metastatic nodules composed of tumor cell clusters and dilated/tortuous vessels could also be observed in the same mice approximately 8 weeks later. 

Previous studies of tumor angiogenesis using multiphoton microscopy have imaged tumor blood vessels by injecting a fluorescent contrast agent [6–8, 13, 14], and blood cells including leukocytes, erythrocytes, and platelets, or endothelial cells could therefore not be visualized. However, blood cells, except for erythrocytes and endothelial cells, can be visualized at higher optical resolutions and at higher magnifications using our TPSLM techniques. The interaction between intravascular cancer cells and endothelial cells or blood cells during the early stage of liver metastasis, as well as metastatic tumor growth with tumor angiogenesis during the late stage of liver metastasis, could therefore both be observed in the same living mice. 

However, this method has several limitations that need to be overcome. The high magnification means that only a narrow observation field can be viewed, which represents only a fraction of the total tumor volume. The frequency of observations that can be made using time-series TPLSM is also limited. In addition, exteriorization of the liver and the development of fibrous adhesions between the abdominal wall and liver surface may affect the physiological condition of the living liver. 

Tumor-host interactions play an important role in tumor metastasis and angiogenesis [1, 15] and are thus a therapeutic target in human cancers [2, 16]. *In vivo* visualization of real-time tumor-host interactions at the cellular or subcellular level with high spatial resolution therefore represents a valuable means of studying tumor angiogenesis in a preclinical tumor model. 

Like a TPLSM, second harmonic generation (SHG) imaging is also a nonlinear microscopy technique which directly visualizes the collagen assembly. Thus, SHG microscopy also becomes an interesting application to study the tumor-stromal interaction by imaging the stromal collagen. In the future study, we need to use SHG imaging as well as TPLSM for analyzing the tumor-host interaction including tumor metastasis and angiogenesis. 

High-resolution, dual-color, *in vivo* real-time visualization of tumor metastasis and angiogenesis using intravital and time-series TLPSM can help to improve our understanding of spatiotemporal tumor-host interactions during metastatic processes in the organs of living animals. It may also provide an ideal method for antiangiogenic drug evaluation, reducing the effects of interindividual variation.

## Figures and Tables

**Table 1:** Early events of colorectal liver metastasis in the portal route model.

Early event	RFP-HT29 cells in GFP nude mice
Tumor-cell arrest/adhesion	20/20* (100%)^#^
Tumor-cell-induced platelet aggregation	15/20 (75%)
Extravasation from hepatic sinusoids	1/20 (5%)
Phagocytosis by Kupffer cells	2/20 (10%)

*Number of mice having the indicated phenomenon/number of mice inoculated with the indicated cells.

^#^Percentage.

**Table 2:** Rate of liver metastasis formation and observation by intravital TPLSM.

	RFP-HT29 cells in GFP nude mice
Liver metastases after intrasplenic inoculation	70%^a^ (12/17)^b^ at 8 weeks
Observation of metastasis by intravital TPLSM	67%^c^ (8/12)^d^ at 8 weeks

^a^Percentage of liver metastases after intrasplenic inoculation.

^b^The number of mice with liver metastases/the number of mice avaiable at the indicated time points.

^c^Percentage of successful observation of metastatic colonies by intravital TPLSM.

^d^The number of mice successfuly observed metastatic colonies by intravital TPLSM/the number of mice with liver metastases.
